# Small Heart Size and Premature Death in 366,484 Individuals With Normal Ejection Fraction

**DOI:** 10.1016/j.jacadv.2024.101444

**Published:** 2024-12-13

**Authors:** Stephanie J. Rowe, Elizabeth D. Paratz, Louise Fahy, Kristel Janssens, Luke W. Spencer, Paolo D’Ambrosio, Geoff Strange, David L. Prior, David Playford, Andre La Gerche

**Affiliations:** a HEART Lab, St Vincent’s Institute of Medical Research, Fitzroy, Australia; b Heart, Exercise and Research Trials, Victor Chang Cardiovascular Research Institute, Darlinghurst, Australia; c Department of Cardiology, St Vincent’s Hospital Melbourne, Fitzroy, Victoria, Australia; d Department of Medicine, University of Melbourne, Parkville, Victoria, Australia; e Sports Cardiology, Baker Heart and Diabetes Institute, Melbourne, Australia; f Exercise and Nutrition Research Program, The Mary MacKillop Institute for Health Research, Australian Catholic University, Melbourne, Australia; g School of Medicine, The University of Notre Dame, Fremantle, Western Australia, Australia

**Keywords:** cardiac size, heart failure, HFpEF, HFrEF

## Abstract

**Background:**

In patients with preserved left ventricular ejection fraction (LVEF), small ventricular size has been associated with reduced functional capacity, but its impact on clinical outcomes is unclear.

**Objectives:**

The goal of this study was to determine the relationship between small heart size and premature mortality within a large multicenter adult patient cohort with transthoracic echocardiographic examinations.

**Methods:**

We divided 366,484 individuals with LVEF ≥50% (including a subset of 279,442 individuals with high-normal LVEF ≥60%) by sex and increasing quartiles for LV end-diastolic volume (LVEDV), LVEDV indexed to body surface area (LVEDVi), and LV end-diastolic diameter to assess associations with 5-year mortality through linkage with the National Death Index.

**Results:**

During approximately 2 million person-years of follow-up, 65,241 deaths occurred. Increasing LV chamber size was associated with reduced odds of 5-year all-cause mortality, particularly for higher LVEF. As compared with the larger quartiles, the smallest cardiac size quartiles were associated with higher 5-year all-cause mortality, even after adjusting for age. The smallest LVEDVi quartile was associated with a 14% to 18% higher odds of 5-year all-cause mortality, with a greater effect with high-normal LVEF. There was a U-shaped relationship between LV chamber size and all-cause mortality. For cardiovascular-related mortality, females in the smallest LVEDVi quartile had a 17% increased odds of mortality, which increased to 30% in those with LVEF ≥60%. In men, there was no significant association between smallest cardiac size and cardiovascular-related mortality.

**Conclusions:**

In individuals with normal LVEF, small ventricular size is associated with increased mortality, particularly among females and those with higher LVEF.

The relationship between cardiac size and clinical outcomes has traditionally focused on the association between larger left ventricular (LV) size and increased mortality. In heart failure (HF) with reduced ejection fraction (HFrEF), LV dilation is a strong predictor of HF hospitalization and mortality.^1^^,^^2^ However, in healthy populations and athletes, LV dilation is associated with improved cardiorespiratory fitness (CRF) and improved longevity.^3^, ^4^, ^5^ At the other end of the spectrum, small heart size has been associated with reduced functional capacity,^6^ but its impact on longevity has not been evaluated.

Functional capacity can be quantified as CRF and is a well-established marker of cardiovascular and all-cause mortality.^7^ Using cardiac magnetic resonance (CMR) imaging during exercise, women with small LV volumes have been shown to have reduced capacity to augment cardiac output (CO), thereby resulting in diminished CRF.^6^ This has not been specifically investigated in men and it may be hypothesized that greater resting cardiac volumes in men provide some protection from functional limitation by this mechanism. Even within the normal range of LV ejection fraction (LVEF), there are likely distinctive phenotypes with clinical and pathophysiological differences between preserved and high-normal LVEF.^8^ There is a potential association between higher LVEF and small cardiac size. This may be due, in part, to the mathematical implications of a normal stroke volume relative to a smaller LV end-diastolic volume (LVEDV). Furthermore, it could represent the fact that a smaller heart may be increasingly reliant on its contractile reserve to maintain adequate output. The clinical consequences of attenuated remodeling resulting in small cardiac size are yet to be determined.

We hypothesized that: 1) small cardiac size would be associated with premature mortality; 2) this association would be stronger in those with high-normal LVEF; and 3) females would be more susceptible than males. Utilizing the National Echocardiography Database of Australia (NEDA), we aimed to describe the profile of cardiac structure and function of males and females with normal LVEF. We then sought to examine the relationship between parameters of LV size and mortality comparing males and females with preserved LVEF (≥50%) and those with high-normal LVEF (≥60%).

## Methods

### Study design

NEDA is a large observational cohort study that captures individual transthoracic echocardiographic data on a retrospective and prospective basis in Australia, the details of which have been described previously.^9^ The study cohort is typically referred for investigation of potential or pre-existing cardiovascular conditions by a general practitioner or cardiologist. For this study, 23 centers throughout Australia contributed data with complete provision of all echocardiographs performed. Participating sites include both public and private centers within Australia’s health care system and therefore NEDA reflects a large heterogenous real-life population. Individual data linkage to the Australian National Death Index (NDI) is then used to derive mortality outcomes. NEDA is registered with the Australian New Zealand Clinical Trials Registry (ACTRN12617001387314). Ethical approval has been obtained from all relevant Human Research Ethics Committees and the study adheres to the Declaration of Helsinki.

### Echocardiographic reports

All echocardiographic measurement and report data, including basic demographic profiling (biological sex and date of birth) and date of investigation collected by participating centers were transferred into a central database via an automated data extraction process during the period January 1, 2000 to May 21, 2019. As a result of this automated process, there is no routine collection of clinical information. All data were then cleaned and transformed into standard NEDA format to remove duplicate, inconsistent, and/or impossible measurements.

**Figure 1:**
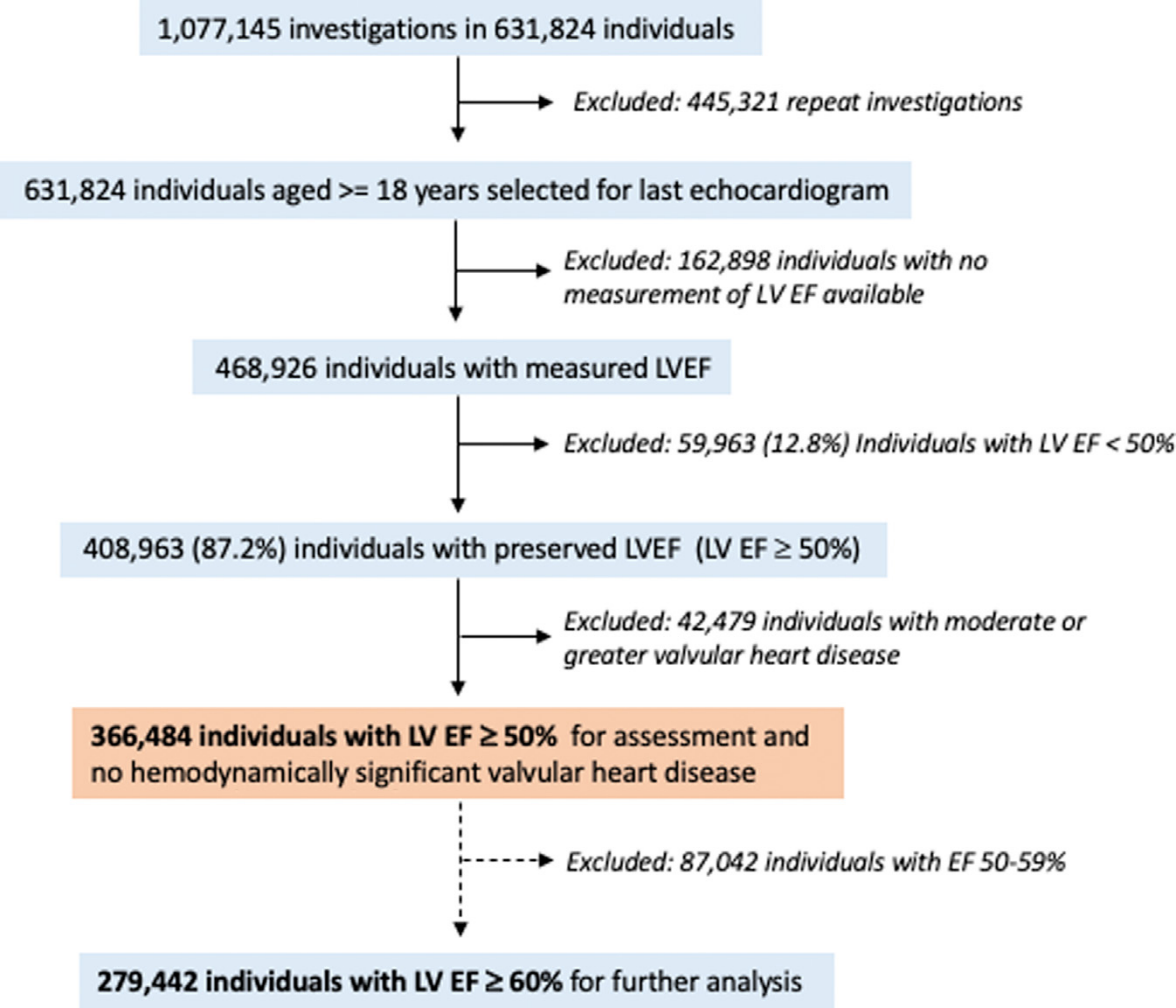
**Study Flowchart** Study flowchart displaying the subjects meeting inclusion criteria for this study. There were 366,484 individuals with a measured LVEF ≥50% and 279,442 individuals with LVEF ≥60% for further subanalysis. LVEF = left ventricular ejection fraction.

For this study, all individuals aged >18 years were selected based on their last reported echocardiogram and the presence of a quantified LVEF. Individuals with reduced LVEF (<50%) and/or moderate or greater valvular disease were excluded (Figure 1). Analyses of LV cardiac chamber size included individuals based on the presence of measured two-dimensional-volumetric LVEDV and/or linear dimensions (LV end-diastolic diameter, measured in the parasternal long axis [LVEDD]).

### Study outcomes

The primary outcomes of this study were all-cause and cardiovascular-related mortality. Survival data were derived from data-linkage performed via Australia’s NDI.^10^ Using a detailed probabilistic matching process, reliable data on survival status up to the study census date of May 21, 2019 were generated. Consistent with previous NEDA analyses,^11^^,^^12^ any listed primary causes of death were categorized according to International Classification of Diseases, Tenth Revision, Australian Modification coding with all chapter codes in the range of I00-I99 categorized as a cardiovascular-related death.

### Statistical analyses

Continuous variables were summarized using median (IQR), and categorical variables using proportions. Mann-Whitney test was used for 2-group comparisons of continuous variables. Individuals were included based on LVEF ≥50%, with a further focused analysis limited to LVEF ≥60%.

For the main analysis of LV chamber size and mortality, individuals were grouped by sex into increasing quartiles based on: 1) LVEDV; 2) body surface area (BSA) indexed LVEDV (LVEDVi); and 3) LVEDD to assess different guideline-directed measures of chamber size.^13^ For 5-year cardiovascular and all-cause mortality, multiple logistic regression was used to generate adjusted ORs (adjORs) and 95% CIs for each cardiac size measure, and for quartiles of LV chamber size relative to the reference group (smallest quartile). Logistic regression models were all adjusted for age, with models assessing differences between quartiles of cardiac size also adjusted for BSA, unless the echocardiographic measure was already indexed to BSA. To assess the mortality outcomes for the smallest cardiac size, adjORs were calculated for 5-year cardiovascular and all-cause mortality comparing the smallest quartile (“small heart”) relative to the “normal” quartiles (2nd, 3rd, and 4th quartile). Violin plots were used to visualize the age distribution for each quartile of LVEDV with Kruskal-Wallis test used for between-group differences. The Kaplan-Meier method followed by Cox-proportional hazard model were used to explore the association between 4 categories of LV size and long-term all-cause mortality. Hazard ratios were adjusted for age and sex. Bland-Altman plots were used to compare the agreement between the Teichholz method (from the LVEDD measurement) and other apical LVEDV methods to assess the reliability of LVEDD as a measure of LV chamber size.

All statistical analyses were performed using STATA, v17.0 (STATACorp). Statistical significance was accepted at a 2-sided α = 0.05.

## Results

### Study cohort

Overall, 185,635 females (50.7%) and 180,849 males with an LVEF ≥50% were studied (n = 366,484), with a median age of 61 years. Table 1 summarizes the baseline echocardiographic profile of the cohort. There were distinct sex differences in measures of cardiac size with females having smaller LV dimensions (*P* < 0.001), volumes (*P* < 0.001), and mass compared to males (*P* < 0.001). LVEDV was smaller in females compared to males even after indexing to BSA (median 41.05 mL/m^2^ [IQR: 33.89-49.00] vs 47.39 mL/m^2^ [IQR: 39.47-56.20] respectively, *P* < 0.001). The proportion of males with LVEF ≥60% as compared with those with LVEF 50 to 60% was less in males than females (46.0% vs 60.1%, *P* < 0.001). Sex-associated differences in cardiac size persisted in those with LVEF ≥60% (Supplemental Table 1).

**Table 1:** Baseline Characteristics and Sex Differences for Individuals With LVEF ≥50%

	All (N = 366,484)	Male (n = 180,849)	Female (n = 185,635)	*P* Value
Age at echo (y)	61 (47-73)	61 (47-72)	62 (47-74)	<0.0001
Body mass index (kg/m^2^)	26.95 (23.77-30.86)	27.34 (22.11-30.69)	26.49 (22.96-31.14)	<0.0001
Body surface area (m^2^)	1.9 (1.72-2.10)	2.01 (1.9-2.18)	1.78 (1.63-1.92)	<0.0001
LA volume (mL)	55 (43-69.79)	60 (47.9-74.10)	50.19 (40-64)	<0.0001
LVEDD (cm)	4.66 (4.30-5.00)	4.87 (4.5-5.2)	4.5 (4.1-4.8)	<0.0001
LVESD (cm)	2.9 (2.5-3.2)	3.00 (2.7-3.3)	2.72 (2.4-3.0)	<0.0001
LVEDV (mL)	83.34 (66.26-104)	96.00 (79-116)	72.6 (59-88.7)	<0.0001
LVEDVi (mL/m^2^)	44.02 (36.3-52.8)	47.39 (39.47-56.20)	41.05 (33.89-49)	<0.0001
LVESV (mL)	33 (24.9-43)	38.6 (30.0-48.5)	28.0 (21.3-36.0)	<0.0001
LVESVi (ml/m^2^)	17.1 (13.2-21.67)	18.82 (14.77-23.42)	15.49 (12.06-19.51)	<0.0001
LVEF (%)	65 (60-70)	63.15 (58.95-69)	65.4 (60.0-71)	<0.0001
LV mass (g)	152.04 (123.3-181.91)	170.19 (145.24-196.12)	135.94 (112.54-163.13)	<0.0001
LVOT SV (mL)	75.7 (62.4-90.85)	82.13 (68.25-97.19)	69.95 (58.2-83.23)	<0.0001

Values are median (IQR).

Age n = 366,484; body mass index n = 256,775; body surface area n = 258,624; LA volume = left atrial volume n = 157,832; LVEDD = left ventricular end diastolic diameter n = 305,771; LVEDV = left ventricular end-diastolic volume n = 140,849; LVEDVi = LV end-diastolic volume indexed to BSA n = 135,974; LVESD = left ventricular end-systolic diameter n = 274,950; LVESV = left ventricular end-systolic volume n = 70,407; LVESVi = LV end-systolic volume indexed to BSA n = 65,921; LVEF = LV ejection fraction n = 366, 484; LV mass n = 268,619; LVOT SV = left ventricular outflow tract stroke volume n = 116,609.

**Figure 2:**
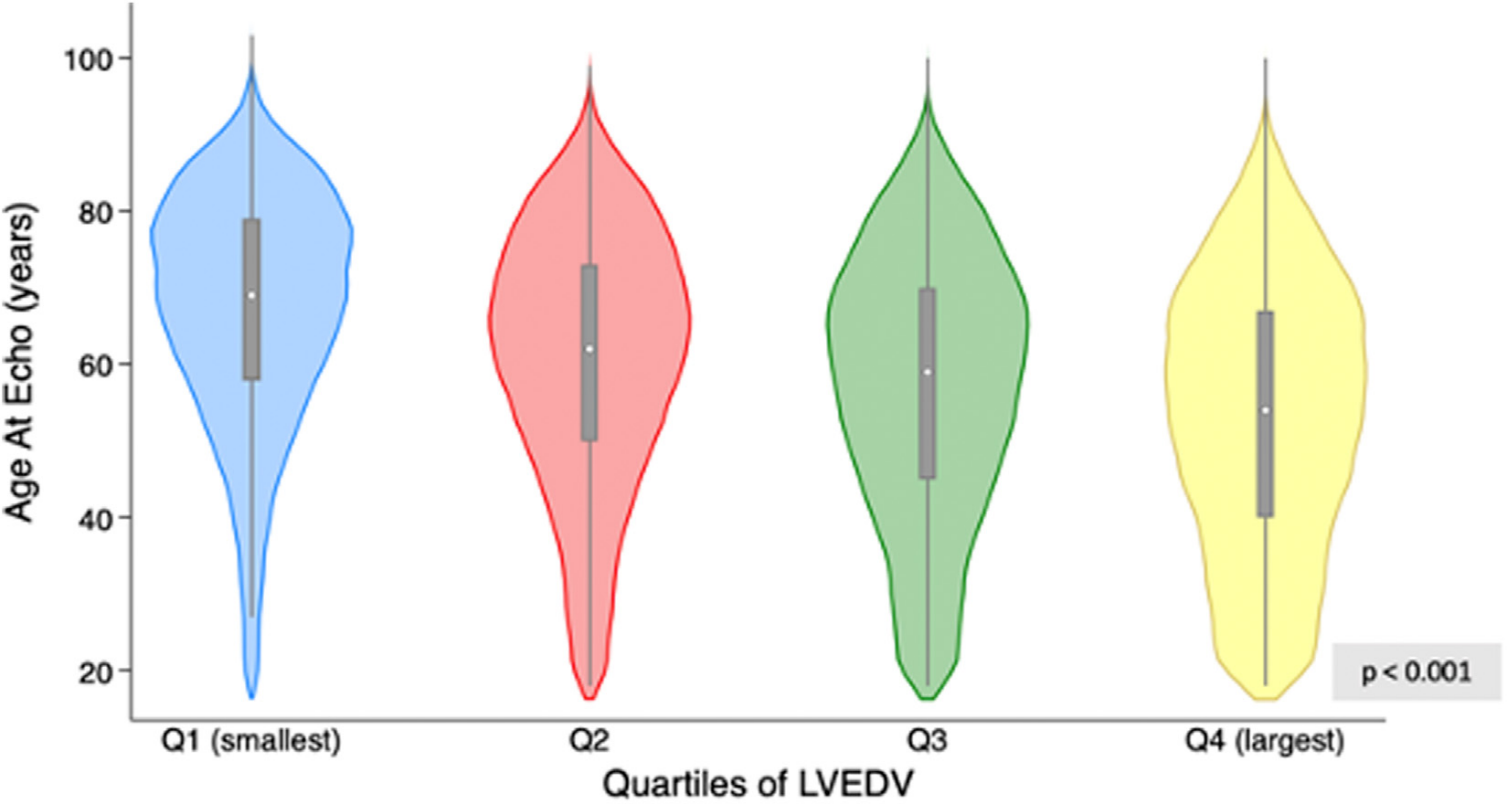
**Distribution of Age at Echo for Increasing Quartiles of Left Ventricular End-Diastolic Volume in Individuals With Left Ventricular Ejection Fraction ≥50%** Violin plots demonstrating median (white dot), IQR (blue boxes), and kernel density estimation showing distribution shape. LVEDV = left ventricular end-diastolic volume; Q = quartile.

Figure 2 demonstrates the relationship between LVEDV and age at echocardiogram, with increasing quartiles of LVEDV associated with a younger age distribution.

### LV volumetric and dimensional quartile definitions

Table 2 demonstrates the cutoff values for LV volumetric and dimensional quartiles by sex based on the 25th, 50th, and 75th percentile of each echocardiographic parameter and LVEF. The lowest quartile (Q1) reflects the smallest values of cardiac chamber size.

**Table 2:** Cutoff Values for Quartiles Based on 25th, 50th, and 75th Percentile for Echocardiographic Parameter by Sex

	Male							
	LVEF ≥50% (n = 180,849)				LVEF ≥60% (n = 128,523)			
	Q1	Q2	Q3	Q4	Q1	Q2	Q3	Q4
LVEDV (mL)	≤79.0	79.0-96.0	96.0-116.0	>116.0	≤77.0	77.0-93.0	93.0-112.0	>112.0
LVEDVi (mL/m^2^)	≤39.5	39.5-47.4	47.4-56.2	>56.2	≤38.5	38.5-46.1	46.1-54.5	>54.5
LVEDD (cm)	≤4.5	4.5-4.9	4.9-5.2	>5.2	≤4.5	4.5-4.8	4.8-5.2	>5.2

	Female							
	LVEF ≥50% (n = 185,635)				LVEF ≥60% (n = 150,919)			
	Q1	Q2	Q3	Q4	Q1	Q2	Q3	Q4
LVEDV (mL)	≤59.0	59.0-72.6	72.6-88.7	>88.7	≤58.0	58.0-71.0	71.0-86.6	>86.6
LVEDVi (mL/m^2^)	≤33.9	33.9-41.1	41.1-49.0	>49.0	≤33.5	33.5-40.3	40.3-48.1	>48.1
LVEDD (cm)	≤4.1	4.1-4.5	4.5-4.8	>4.8	≤4.1	4.1-4.5	4.5-4.8	>4.8

Q1 = quartile 1 (smallest measures); Q2 = quartile 2; Q3 = quartile 3; Q4 = quartile 4 (largest measures); other abbreviations as in Table 1.

### The ‘small heart’-small cardiac size and mortality

Among the 366,484 individuals with measured LVEF ≥50%, there were a total of 65,241 (17.8%) all-cause deaths during 2 million person-years of follow-up. Overall, 47.3% of these deaths were females. Cardiovascular-related mortality accounted for 15,300 (23.5%) of all deaths. Of all measures of cardiac chamber size, LVEDV was recorded in 140,849 (38.4%) and LVEDD was recorded in 305,771 (83.4%) individuals. Supplemental Figure 1 shows the extent of agreement between Teichholz, Method of Discs, and Area-Length measures of LVEDV using Bland-Altman plots to assess the reliability of LVEDD as a measure of LV chamber size. There was a moderate amount of variability between the Teichholz estimates and volumetric measures, with the best agreement with smaller LV size.

**Figure 3:**
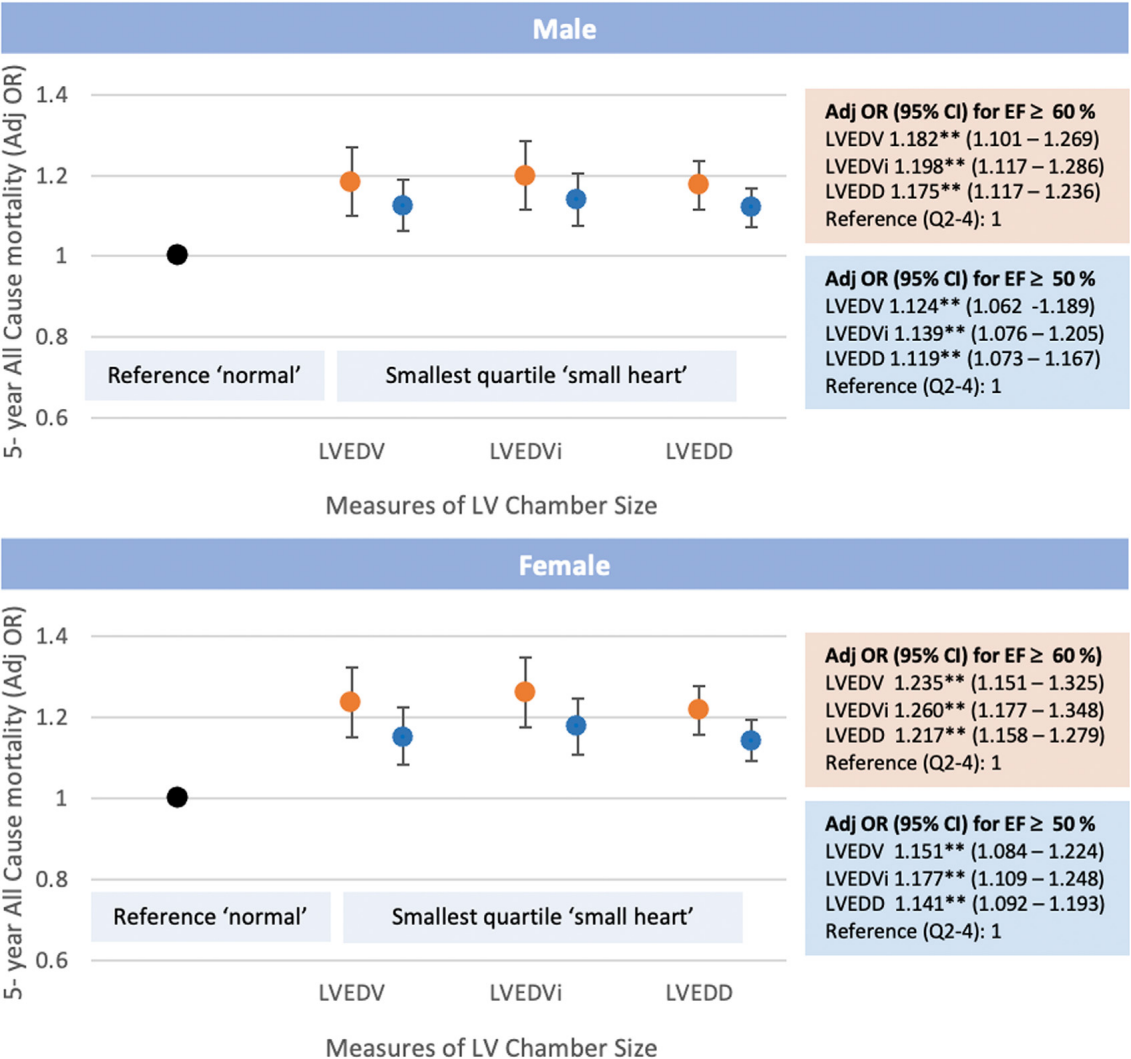
**The “Small Heart: 5-Year All-Cause Mortality for Smallest Cardiac Size Compared to Reference “Normal” for Males and Females** Plots show the adjusted OR (±95% CI) for all-cause mortality for the smallest quartile of cardiac size relative to the reference group (remaining quartiles) for individuals with LVEF ≥50% (blue circle) and LVEF ≥60% (orange circle). The box inserts show the adjusted ORs (±95% CI). Adj OR = OR adjusted for age and BSA unless already indexed to BSA. The significance for each OR is denoted by ∗∗*P* < 0.0001. LVEDD = left ventricular end-diastolic diameter; LVEDVi = LVEDV indexed to BSA; other abbreviation as in Figure 2.

Figure 3 shows the age- and BSA-adjusted odds ratio for 5-year all-cause mortality for individuals with chamber sizes in the smallest quartile for cardiac size, relative to the remaining quartiles. Overall, the smallest measures of LVEDV, LVEDVi, and LVEDD were associated with poorer 5-year survival in both males and females. For all measures of cardiac chamber size, the lowest quartile in individuals with LVEF ≥50% was associated with a ∼12% to 14% and 14% to 17% increased odds of 5-year all-cause mortality for males and females, respectively. For high-normal LVEF (LVEF ≥60%), these odds were increased to 18% to 20% for males and 21 to 26% for females. This lowest quartile, termed the “small heart,” corresponds to a measure of LVEDV of ≤77 mL and LVEDVi of ≤38.5 mL/m^2^ for males, and an LVEDV of ≤58 mL and LVEDVi of ≤33.5 mL/m^2^ in females (Table 2).

A “small heart” by volumetric parameters was associated with a 30% higher odds of 5-year cardiovascular-related mortality in females with higher LVEF (≥60%). No such association was found in males (Supplemental Figure 2).

### Cardiac chamber size and all-cause mortality

**Figure 4:**
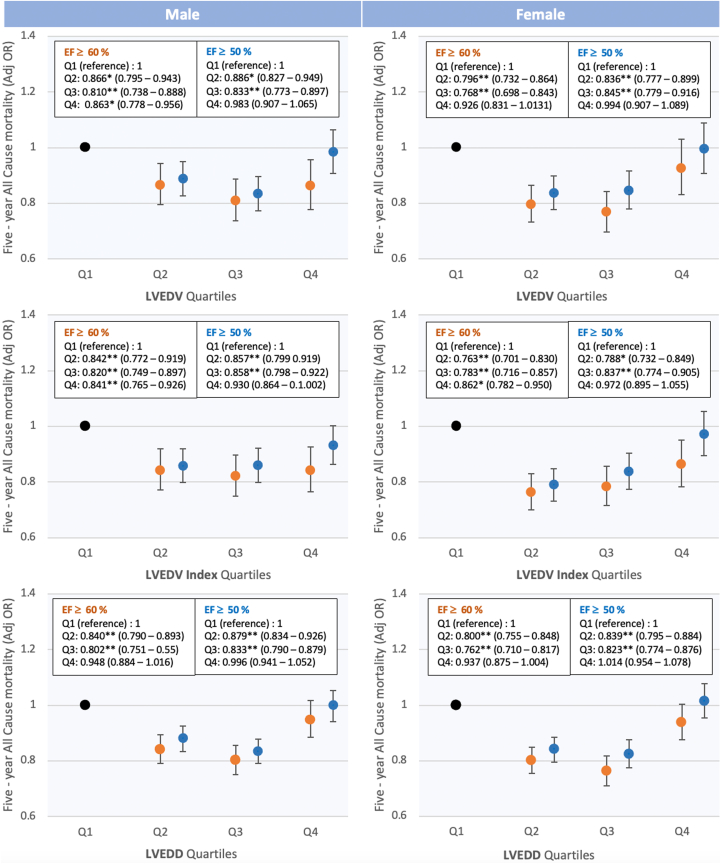
**5-Year Adjusted All-Cause Mortality by Sex and Quartiles of Cardiac Chamber Measures** The box inserts show the adjusted odds ratios (± 95% CI). Plots show the adjusted odds ratio (±95% CI) for all-cause mortality for increasing quartiles of cardiac size relative to the smallest quartile for individuals with LVEF ≥50% (blue circle) and LVEF ≥60% (orange circle). Q = quartile; Adj OR = OR adjusted for age and BSA unless already indexed to BSA. ∗*P* < 0.05 and ∗∗*P* < 0.0001. Abbreviations as in Figures 2 and 3.

Figure 4 summarizes the 5-year survival profile of the cohort by sex according to increasing quartiles of LVEDV, LVEDVi, and LVEDD for all-cause mortality and adjusted for age and BSA.

There was a U-shaped relationship between LV chamber size and all-cause mortality. In both males and females, compared to the smallest quartile across all measures of LV cardiac chamber size, cardiac size between the 25th–75th percentile were associated with lower 5-year all-cause mortality. This was most profound for individuals with LVEF ≥60% where cardiac size measurements in the 2nd or 3rd quartiles were associated with odds of all-cause mortality at 5 years that were 14% to 19% lower in males, and 20% to 24% lower in females. The highest quartile of LV chamber measures showed no significant difference in all-cause mortality compared to the smallest quartile for all with LVEF ≥50%. For high-normal LVEF, LV volumetric measures in the highest quartile were associated with lower odds of 5-year all-cause mortality in males; however in females, this was only true when LVEDV was BSA-indexed (LVEDV adjOR: 0.926 [95% CI: 0.831-1.013, *P* > 0.05], LVEDVi adjOR: 0.862 [95% CI: 0.782-0.950, *P* < 0.05]).

Eight continuous parameters of cardiac size and function were assessed with respect to 5-year all-cause mortality (Supplemental Table 2). Of these parameters and after adjusting for age, increasing LV volumetric and linear dimensions were associated with lower odds of 5-year all-cause mortality, particularly in those with LVEF ≥60%. In individuals with LVEF ≥50%, the adjusted odds ratio for 5-year all-cause mortality for increasing LVEDD was 0.815 (95% CI: 0.792-0.838, *P* < 0.001) in males and 0.839 (95% CI: 0.813-0.867, *P* < 0.001) in females. The protective effect of increasing LVEDD was even greater in those with LVEF ≥60%; 0.765 (95% CI: 0.740-0.792, *P* < 0.001) for males and 0.772 (95% CI: 0.745-0.800, *P* < 0.001) for females.

#### Cardiac chamber size and cardiovascular-related mortality

Supplemental Figure 3 summarizes the 5-year survival profile of the cohort by sex according to increasing quartiles of LVEDV, LVEDVi, and LVEDD for cardiovascular-related mortality and adjusted for age and BSA.

The overall pattern for 5-year cardiovascular-related mortality across the different quartiles was differed between sexes. Relative to the smallest quartile of LV chamber measures, there was no significant difference in the odds of 5-year cardiovascular mortality with measures in the 2nd or 3rd quartile for males. However, measures of LVEDV, LVEDVi, and LVEDD in the highest quartile were associated with 18% to 36% higher odds of cardiovascular mortality at 5 years in males with LVEF ≥50%. In females, there was a trend toward this pattern of increased risk with LV measures in the highest quartile, however the odds of cardiovascular mortality differed for volumetric and linear measures depending upon the LVEF cutoff (for LVEF ≥60%: LVEDVi adjOR: 0.794 [95% CI: 0.648-0.973, *P* < 0.05] vs LVEDD adjOR: 1.145 [95% CI: 1.001-1.309, *P* < 0.05]). Similar to all-cause mortality, females with LVEF ≥60% and LV volumetric measures in the 2nd or 3rd quartile had reduced odds of 5-year cardiovascular-related mortality compared to the smallest quartile, with LV volumetric measures in the 3rd quartile associated with a reduction in odds of cardiovascular mortality between 27% and 37%.

Supplemental Table 3 shows the results for continuous parameters of cardiac size and function and 5-year cardiovascular-related mortality adjusted for age. Findings for cardiovascular mortality and increasing LVEDD were similar to those of all-cause mortality for individuals with LVEF ≥60%, however when LVEF criteria were expanded to ≥50%, there was no longer a significant relationship with cardiovascular mortality. Findings for volumetric measures differed by sex. There was no significant relationship between LV volumetric measures and 5-year cardiovascular mortality in males, however increasing LVEDV and LVEDVi in females was associated with lower odds of 5-year cardiovascular mortality in individuals with LVEF ≥60% (LVEDV adjOR: 0.992 [95% CI: 0.989-0.996, *P* < 0.0001], LVEDVi adjOR: 0.991 [95% CI: 0.984-0.997, *P* = 0.006]. Increasing LV mass was associated with higher odds of 5-year cardiovascular mortality for both sexes (*P* < 0.0001), however increasing LV mass was associated with lower odds of 5-year all-cause mortality in males.

### Cardiac chamber size and LONG-TERM all-cause mortality

**Figure 5:**
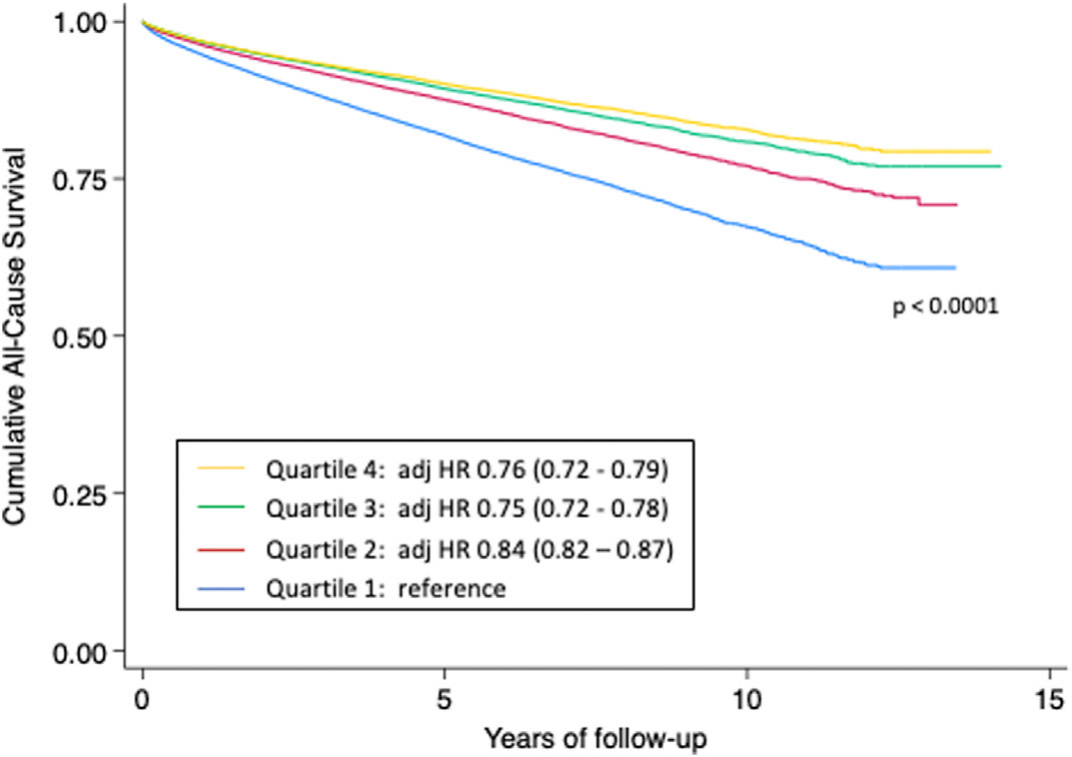
**Long-Term All-Cause Mortality by Left Ventricular End-Diastolic Volume Quartiles** The Kaplan-Meier curve shows long-term all-cause mortality for individuals with LVEF ≥50%. Box insert shows HRs and 95% CIs adjusted for age and sex for quartiles of cardiac size relative to the smallest quartile (quartile 1).

Figure 5 shows long-term all-cause mortality by quartiles of LVEDV for individuals with LVEF ≥50% and hazard ratios adjusted for age and sex. This pattern demonstrates that an LVEDV in the lower quartiles is associated with worse long-term survival.

**Central Illustration:**
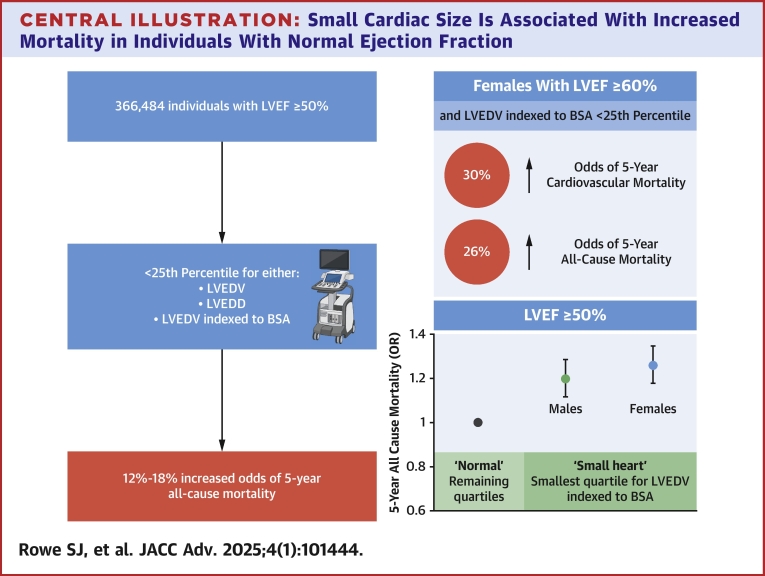
**Small Cardiac Size Is Associated With Increased Mortality in Individuals With Normal Ejection Fraction** This figure was Created using biorender.com. BSA = body surface area; OR = odds ratio adjusted for age; other abbreviations as in Figure 1, Figure 2, Figure 3.

## Discussion

In the largest ever study linking echocardiographic parameters and clinical outcomes for individuals with normal LVEF, we demonstrate a novel association between small cardiac size and increased all-cause and cardiovascular mortality. Utilizing the large and extensive NEDA cohort, echocardiographic data were analyzed from 366,484 individuals across the spectrum of normal LVEF. In those individuals with cardiac volumes or dimensions in the lowest quartile, termed the “small heart,” individuals with high-normal LVEF (≥60%) had an 18% to 26% greater odds of 5-year all-cause mortality even after adjusting for age and BSA (Figure 3). Similar findings were found when the population of interest was expanded to include all subjects with LVEF ≥50% although the magnitude of effect was less. The impact of small LV chamber size on cardiovascular mortality differed between sexes. Females with the smallest LV volumetric measures had a 30% higher odds of 5-year cardiovascular mortality compared to other quartiles, however this was not seen in males.

In recent years, small heart size in those with normal LVEF has been linked with both increased LV diastolic stiffness and low CRF^14^^,^^15^ suggesting that this phenotype may be an important risk factor for developing HF with preserved ejection fraction (HFpEF).^5^^,^^16^ Driven by the inability to augment CO in response to physical demands,^6^ smaller hearts appear to have less capacity to deal with illness or external demands, resulting in an inherent vulnerability for these patients. This is one mechanism by which our study findings of increased all-cause and cardiovascular mortality with small heart size may be explained.

The association between all-cause mortality and quartiles of LV size (Figure 4) suggests a nonlinear “U-shaped” relationship that differs according to the LVEF cutoff used. In both males and females, worse outcomes were seen in those with the smallest and the largest cardiac size. In a CMR study of the Multi-Ethnic Study of Atherosclerosis cohort, LV dilation (considered an LVEDD >95th percentile) was associated with incident HF irrespective of LVEF.^2^ During 12 years of follow-up in the Multi-Ethnic Study of Atherosclerosis study, 71% of incident HF events were HFrEF. The association between higher mortality and largest cardiac size quartile in our study likely reflects a similar phenomenon. Consistent with our hypothesis and contrary to traditional thinking of myocardial thickening associated with mortality, we show an initially counterintuitive concept that higher mass is associated with lower all-cause mortality in men (Supplemental Table 2). However, this may be driven by the fact that larger cardiac volumes are associated with greater LV mass as opposed to referring to thickness.

Importantly, sex differences in the relationship between the “small heart” and 5-year cardiovascular-related mortality (Supplemental Figure 2) may reflect the differing HF phenotypes between men and women as the risk of HFrEF is much lower in women, and female sex is a key clinical discriminating feature between HFpEF vs HFrEF.^17^^,^^18^ Furthermore, the relationship between small cardiac size and older age (Figure 2) may be one mechanism by which the risk of HFpEF increases with age.^18^ Irrespective of the diagnosis of HFpEF, our results demonstrate that the relationship between cardiac size and mortality differs based on what is considered a “normal” or “preserved” LVEF as well as sex. There is increasing evidence that stratifying patients with HFpEF based on LVEF identifies distinct subphenotypes which vary in pathophysiology, morphology and treatment response.^19^^,^^20^ Half of all patients with HF are labeled as preserved LVEF (≥50%), however recent NEDA reports show clinically relevant sex-specific differences in mortality within this group based on further categorization of LVEF.^21^^,^^22^ This suggests a nadir in mortality risk higher than traditional reference ranges and highlights the increased mortality associated with higher LVEF. Our data extend this concept by highlighting the influence of small cardiac size on mortality that is then further accentuated by higher LVEF. Thus, subcategorization of this higher LVEF cohort according to LV chamber size provides valuable risk stratification of this heterogenous patient group.

The latest referenced American Society of Echocardiography Guidelines for Chamber Quantification (2015) do not provide a cutoff for abnormally small volumes and dimensions,^13^ however having established a subpopulation in whom small cardiac size is associated with premature mortality it is important to define normal lower limits to cardiac chamber size as well as upper limits in future guidelines. While identifying sex-specific cutoff values for a small ventricle was not the primary aim of this current study, we found that the smallest quartile—“small heart”—consisted of males with LVEDV ≤77 ml, LVEDVi ≤38.5 ml/m^2^, or LVEDD ≤4.5 cm and females with LVEDV ≤58 ml, LVEDVi ≤33.5 ml/m^2^, and LVEDD ≤4.1 cm for LVEF ≥60%. Interestingly, these are *smaller* than “abnormally small” LV volumes quoted in CMR references^23^ but perhaps not too dissimilar when one considers that CMR volumes are frequently around 20% greater than those acquired by two-dimensional echocardiography.^24^^,^^25^ Combining the prognostic significance of the small heart with the knowledge that small ventricular size is associated with impaired CRF, it would seem prudent that the concept of the small ventricle is considered an at-risk phenotype, particularly in females.

Lastly, this study invites speculation as to whether the small heart phenotype, characterized by small volumes and higher LVEF, is likely to be a cause of HF. Small cardiac volumes have been associated with lesser CO under physical stress^6^ which may result in a low-output failure that may be more characterized by organ hypoperfusion during intercurrent illness rather than by raised cardiac filling pressures. Consequently, this phenotype may be missed by some contemporary definitions of HF that rely on findings of pulmonary congestion, raised filling pressure, and/or raised natriuretic peptides.^26^ Our findings raise the question as to whether the definition of HF may need to be broadened to consider this entity. This is especially pertinent given that it may be a phenotype of HFpEF with a very specific treatment. Exercise has been shown to increase cardiac size and functional capacity. Awareness of the small heart phenotype may identify an important population in whom interventional trials (including structured exercise programs) could be aimed.

### Study limitations

Previous NEDA reports have outlined the common limitations associated with using and interpreting big data.^12^^,^^27^ It is particularly relevant to this study that clinical details such as HF diagnosis or symptomatology are not captured by NEDA and so we do not know if patients may have had a clinical syndrome of HFpEF. In addition, cardiac size and remodeling may be influenced by many modifiable and nonmodifiable factors. Differences in cardiac size with sex and age were investigated and adjusted for in this study, however additional factors influencing cardiac size and cardiovascular risk, such as smoking, ethnicity and levels of regular physical activity, were not available for assessment. This study assessed 3 key and commonly utilized measures of LV cavity size, with the current recommended technique being LVEDVi.^13^ However, across this large nationwide study, this parameter was only measured in 37% of individuals. This may reflect time constraints in clinical practice impacting volumetric assessment or possibly a bias toward linear measures when LVEF is within normal limits due to the lack of clinical implications behind cardiac size in this population. Additionally, 25% of participants with small LVEDV were categorized in quartile 2 for LVEDD which may have a prognostic impact and highlights discrepancies between linear and volumetric measures. Despite this, we were able to consistently demonstrate a similar pattern of mortality utilizing different parameters of LV cavity size. Although explored, in-depth analysis of the relationship between mortality and additional echocardiographic variables such as LV mass was not within the scope of this study. Lastly, mortality data have been derived from the NDI of Australia and thus the reliability of cardiovascular-related mortality, as opposed to all-cause mortality, depends on the appropriate coding of death certificates.

## Conclusions

In individuals with a normal ejection fraction, there is a subset of patients with small cardiac size who are at greater risk of mortality, particularly in women with higher LVEF. Small cardiac size has previously been associated with reduced functional capacity and we now demonstrate an association between cardiac size and premature cardiovascular and all-cause mortality. This supports the concept of varied phenotypes within the large majority of the population who have a normal ejection fraction. Much attention has been paid to hearts of larger size and those with LVEF in the lower normal range. Our data invite scientific focus on the other end of the spectrum, those with higher ejection fractions and smaller cardiac volumes in whom the prognosis may be similarly guarded.

Perspectives **COMPETENCY IN MEDICAL KNOWLEDGE:** Patients with small cardiac chamber size in the setting of a normal ejection fraction are at increased risk of mortality, particularly among females and higher ejection fraction.

**TRANSLATIONAL OUTLOOK 1:** The determinants of the small heart and the mechanism by which it leads to increased mortality remain unknown and require additional research.

**TRANSLATIONAL OUTLOOK 2:** Given exercise increases cardiac size and functional capacity, regular participation may prevent or augment the mortality risk associated with the small heart. Awareness, leading to diagnosis of the small heart, may identify an important population in whom interventional trials could be aimed.

## Funding support and author disclosures

Dr Rowe is supported by the 10.13039/501100000925National Health and Medical Research Council Postgraduate Scholarship, the National Heart Foundation PhD scholarship and the Elizabeth and Vernon Puzey Scholarship. Prof La Gerche is supported by a 10.13039/501100000925National Health and Medical Research Council Investigator Grant (Level 1, APP#2027105). All other authors have reported that they have no relationships relevant to the contents of this paper to disclose.
