# Supplementary material for: Small Heart Size and Premature Death in 366,484 Individuals With Normal Ejection Fraction
Source: JACC Adv. 2024 Dec 13;4(1):101444. doi: 10.1016/j.jacadv.2024.101444 (PMC11699313; doi:10.1016/j.jacadv.2024.101444)
Supplement: Supplemental material [file mmc1.docx]

**Supplemental Figure 1.** *Bland-Altman Plots.***
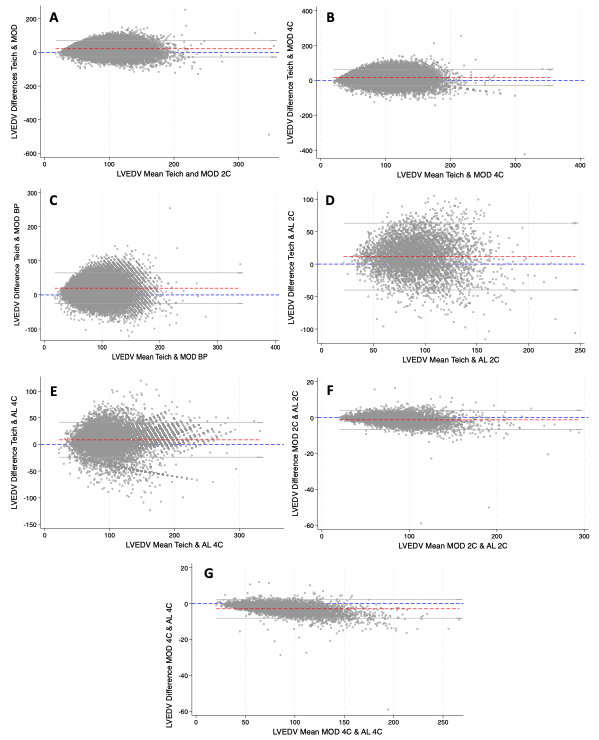
**

The means of 2 methods are plotted against the difference between the 2 measurements to analyse the agreement between different estimates of left ventricular chamber size. The closest agreement occurs with smaller chamber size. Red line = mean difference. MOD BP = Biplane, method of discs; Teich = Teicholz Method; AL = Area-length method; 2C = 2 chamber; 4C = 4 chamber.

**Supplemental Figure 2.** *The ‘small heart’ - Five-year cardiovascular (CV) mortality for smallest cardiac size compared to reference ‘normal’ for males and females.*

**
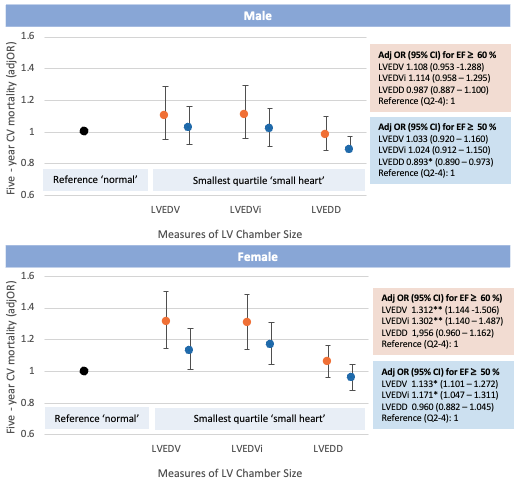
**

Plots show the adjusted odds ratio (+/- 95% CI) for cardiovascular mortality for the smallest quartile of cardiac size relative to the reference group (remaining quartiles) for individuals with LVEF ≥50% (blue circle) and LVEF ≥60% (orange circle). The box inserts show the adjusted odds ratios (+/-95% CI). Adj OR = odd ratio adjusted for age and BSA unless already indexed to BSA; CI = confidence interval. The significance for each odds ratio is denoted by **P < 0.0001. LVEDV = left ventricular end-diastolic volume; LVEDVi = LVEDV indexed to BSA; LVEDD = left ventricular end-diastolic diameter

**Supplemental Figure 3.** *Five-year Adjusted Cardiovascular Mortality by sex and quartiles of cardiac chamber measures.*

**
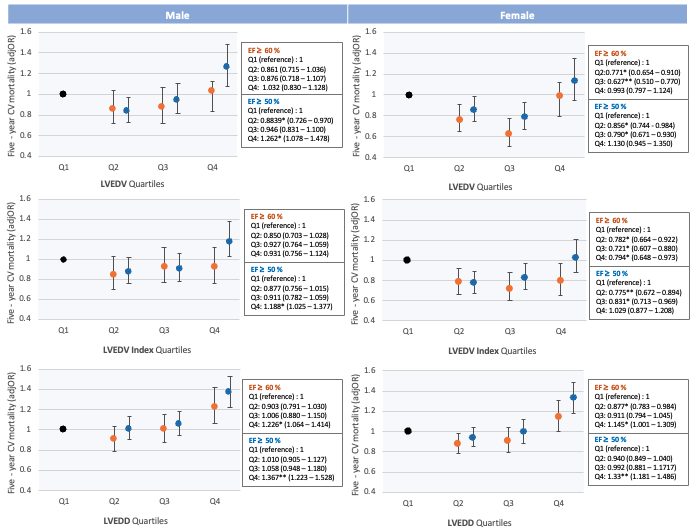
**The box inserts show the adjusted odds ratios (+/- 95% CI). Plots show the adjusted odds ratio (+/- 95% CI) for cardiovascular mortality for increasing quartiles of cardiac size relative to the smallest quartile for individuals with LVEF ≥50 % (blue circle) and LVEF ≥60% (orange circle). Q = quartile; Adj OR = odds ratio adjusted for age and BSA unless already indexed to BSA; CI = confidence interval. LVEDV = left ventricular end-diastolic volume; LVEDVi = LVEDV indexed to body surface area; LVEDD = left ventricular end-diastolic diameter; * P <0.05 and **P < 0.0001.

**Supplemental Table 1.** Baseline Characteristics and sex differences for individuals

with LVEF ≥60%

|  | **All**  (n = 279,442) | **Male**  (n= 128,523) | **Female**  (n= 150,919) | **P-value** |
| --- | --- | --- | --- | --- |
| Age at echo (yrs) | 61  [47-73] | 61  [47-72] | 62  [47-74] | <0.0001 |
| Body Mass Index (kg/ m^2^) | 26.9  [23.7-30.8] | 27.3  [24.5-30.7] | 26.5  [23.0-31.1] | <0.0001 |
| Body Surface Area (m^2^) | 1.9  [1.7-2.1] | 2.0  [1.8-2.2] | 1.78  [1.62-1.91] | <0.0001 |
| LA Volume (ml) | 54  [42-68] | 59  [47-73] | 50  [40-63] | <0.0001 |
| LVEDD (cm) | 4.6  [4.2-5] | 4.8  [4.47-5.2] | 4.5  [4.1-4.8] | <0.0001 |
| LVESD (cm) | 2.8  [2.5-3.1] | 2.91  [2.6-3.2] | 2.7  [2.4-3] | <0.0001 |
| LVEDV (ml) | 80.2  [64-99.7] | 93  [77-112 | 71  [58-86.6] | <0.0001 |
| LVEDVi (ml/m^2^) | 42.8  [35.4-51.2] | 46.1  [38.5-54.5] | 40.3  (33.5-48.1 | <0.0001 |
| LVESV (ml) | 29.1  [22.5-37.3] | 34  [27-42.0] | 25.8  (20-32.3] | <0.0001 |
| LVESVi (ml/m^2^) | 15.4  [12.2-19.1] | 16.7  [13.4-20.4] | 14.3  [11.4-17. | <0.0001 |
| LVEF (%) | 67  [63-71.7] | 66  [63-71] | 67.5  [64-72] | <0.0001 |
| LV Mass (g) | 153.4  [124.5-188.1] | 177.0  [148.1-210.1] | 137.2  [113.6-165.0] | <0.0001 |
| LVOT SV (ml) | 76.2  [63.0-91.4] | 83.3  [69.3-98.6] | 70.7  [59.0-84.2] | <0.0001 |

Legend: Values are median +/- IQR or %. Age n = 279,442 ; body mass index n = 196,204; body surface area n = 197,381; left atrial volume (LA volume) n = 119,110; left ventricular end diastolic diameter (LVEDD) n = 236,293; left ventricular end-systolic diameter (LVESD) n= 221,029; left ventricular end-diastolic volume (LVEDV) n= 103,539; left ventricular end-diastolic volume indexed to BSA (LVEDVi n= 100,282; left ventricular end-systolic volume (LVESV) n =48,692; left ventricular end-systolic volume indexed to BSA (LVESVi) n = 45,856; left ventricular ejection fraction (LVEF) n =279,442; LV mass n= 226,793; Left ventricular outflow tract Stroke Volume (LVOT SV) n = 80,818

**Supplemental Table 2.** Five- year Adjusted All-cause Mortality and Continuous Measures of Cardiac Size by Sex

|  | **Male** | | | | **Female** | | | |
| --- | --- | --- | --- | --- | --- | --- | --- | --- |
|  | **LVEF ≥ 50%** | | **LVEF ≥ 60%** | | **LVEF ≥ 50%** | | **LVEF ≥ 60%** | |
|  | **Adj OR**  **(95% CI)** | **P-value** | **Adj OR**  **(95% CI)** | **P- value** | **Adj OR**  **(95% CI)** | **P-value** | **Adj OR**  **(95% CI)** | **P- value** |
| LVEDV  (ml) | 0.997  (0.996-0.998) | <0.0001 | 0.995  (0.993 – 0.996) | <0.0001 | 0.995  (0.994-0.997) | <0.0001 | 0.993  (0.991 – 0.995) | <0.0001 |
| LVEDVi  (ml/m^2^) | 0.997  (0.995 – 0.999) | 0.005 | 0.993  (0.990 - 0.996) | <0.0001 | 0.997  (0.994 - 0.9998) | 0.039 | 0.993  (0.990 -0.996) | <0.0001 |
| LVEDD  (cm) | 0.815  (0..792 – 0.838) | <0.0001 | 0.765  (0.740 – 0.792) | <0.0001 | 0.839  (0.813 – 0.867) | <0.0001 | 0.772  (0.745 - 0.800) | <0.0001 |
| LVESD  (cm) | 0.936  (0.903 -0.970) | <0.0001 | 0.830  (0.796 – 0.865) | <0.0001 | 1.023  (0.984 – 1.064) | 0.254 | 0.848  (0.810 – 0.887) | <0.0001 |
| LVEF  (%) | 0.993  (0.991 -0.995) | <0.0001 | 1.005  (1.002 – 1.007) | 0.001 | 0.990  (0.989 – 0.992) | <0.0001 | 1.004  (1.001 - 1.007) | 0.002 |
| LV mass  (g) | 0.998  (0.997 – 0.998) | <0.0001 | 0.999  (0.999 – 0.9999) | 0.026 | 1.001  (1.000 – 1.002) | <0.0001 | 1.002  (1.001 – 1.002) | <0.0001 |
| LVESV  (ml) | 0.991  (0.989-0.994) | <0.0001 | 0.981  (0.977 – 0.986) | <0.0001 | 0.993  (0.989 – 0.996) | <0.0001 | 0.984  (0.978 – 0.989) | <0.0001 |
| LVESVi  (ml/m^2^) | 0.987  (0.981 – 0.993) | < 0.0001 | 0.969  (0.960 – 0.979) | <0.0001 | 0.99995  (0.993 – 1.007) | 0.989 | 0.984  (0.973 – 0.995) | 0.003 |

**Legend:** Adj OR = odds ratio adjusted for age; CI = confidence interval; LVEDV= left ventricular end-diastolic volume; LVEDVi = LVEDV indexed to body surface area; LVEDD =left ventricular end diastolic diameter; LVESD = left ventricular end-systolic diameter; LVEF = LV ejection fraction; LVESV = left ventricular end-systolic volume; LVESVi = LV end-systolic volume indexed to BSA

**Supplemental Table 3.** Five- year Adjusted Cardiovascular-related Mortality and Continuous Measures of Cardiac Size by Sex

|  | **Male** | | | | **Female** | | | |
| --- | --- | --- | --- | --- | --- | --- | --- | --- |
|  | **LVEF ≥ 50%** | | **LVEF ≥ 60%** | | **LVEF ≥ 50%** | | **LVEF ≥ 60%** | |
|  | **Adj OR**  **(95% CI)** | **P-value** | **Adj OR**  **(95% CI)** | **P- value** | **Adj OR**  **(95% CI)** | **P-value** | **Adj OR**  **(95% CI)** | **P- value** |
| LVEDV  (ml) | 1.001  (0.9991- 1.000) | 0.253 | 0.998  (0.995 – 1.001) | 0.143 | 0.996  (0.994 – 0.999) | 0.007 | 0.992  (0.989 – 0.996) | <0.0001 |
| LVEDVi  (ml/m^2^) | 1.004  (0.9996 – 1.009) | 0.073 | 0.995  (0.989 -1.002) | 0.153 | 0.998  (0.993 – 1.004) | 0.533 | 0.991  (0.984 – 0.997) | 0.006 |
| LVEDD  (cm) | 1.019  (0.964 – 1.077) | 0.500 | 0.918  (0.858 – 0.982) | 0.014 | 1.021  (0.962 -1.083) | 0.501 | 0.899  (0.840 – 0.962) | 0.002 |
| LVESD  (cm) | 1.182  (1.100 – 1.270) | <0.0001 | 1.017  (0.935 – 1.105) | 0.699 | 1.217  (1.132 -1.309) | <0.0001 | 0.949  (0.872 – 1.033) | 0.231 |
| LVEF  (%) | 0.983  (0.9796 – 0.987) | <0.0001 | 1.003  (0.998 - 1.008) | 0.278 | 0.987  (0.984 – 0.990) | <0.0001 | 1.006  (1.001 – 1.010) | 0.012 |
| LV mass  (g) | 1.003  (1.002 – 1.004) | <0.0001 | 1.004  (1.003 – 1.005) | <0.0001 | 1.006  (1.005 – 1.007) | <0.0001 | 1.005  (1.005 – 1.006) | <0.0001 |
| LVESV  (ml) | 1.004  (0.999 – 1.01) | 0.118 | 0.991  (0.982 – 0.999) | 0.041 | 1.001  (0.993 - 1.008) | 0.818 | 0.986  (0.975 - 0.997) | 0.015 |
| LVESVi  (ml/m^2^) | 1.009  (0.997 – 1.021) | 0.136 | 0.982  (0.962 – 1.00) | 0.081 | 1.013  (0.998 – 1.027) | 0.086 | 0.988  (0.967 – 1.010) | 0.285 |

Legend: Adj OR = odds ratio adjusted for age; CI = confidence interval; LVEDV= left ventricular end-diastolic volume; LVEDVi = LVEDV indexed to body surface area; LVEDD =left ventricular end diastolic diameter; LVESD = left ventricular end-systolic diameter; LVEF = LV ejection fraction; LVESV = left ventricular end-systolic volume; LVESVi = LV end-systolic volume indexed to BSA
